# School Nurses’ Perceptions, Knowledge, and Related Factors Associated with Evidence-Based Practice in Taiwan

**DOI:** 10.3390/ijerph15091845

**Published:** 2018-08-27

**Authors:** Pei-Lin Hsieh, Sue-Hsien Chen, Li-Chun Chang

**Affiliations:** 1 School of Nursing, Chang Gung University of Science and Technology, Taoyuan City 333, Taiwan; plhsieh@mail.cgust.edu.tw; 2 Chang Gung Memorial Foundation, Taoyuan City 333, Taiwan; 3 Department of Nursing Management, Chang Gung Memorial Foundation Administration Center, Taoyuan City 333, Taiwan; a66998@adm.cgmh.org.tw

**Keywords:** evidence-based practice, school nurse, perception, knowledge, influencing factors

## Abstract

The implementation of evidence-based practice (EBP) in health care has been focused mainly on hospital settings and there is little research on EBP adoption and implementation among school nurses in Taiwan. This study aimed to determine primary school nurses’ perceptions regarding EBP and to explore the factors that influence EBP in Taiwan. A cross-sectional design was used and the School Nurse Evidence-Based Practice Questionnaire was developed to collect data. A total of 2679 elementary school nurses in Taiwan were invited to participate in this study, and 1200 completed questionnaires were returned, with a 45% response rate. Participants’ mean age was 37.5 (range: 31–62) years and the mean duration of employment as a school nurse was 8.4 (range: 1–20) years. The majority of participants had a Bachelor’s degree (56%). The results revealed that participants had insufficient EBP knowledge and skills. However, they had a positive attitude toward and moderate self-efficacy in EBP. The demographic data positively correlated with knowledge, attitudes, skills, self-efficacy, and influencing factors. Certain influencing factors were highlighted. As school nurses play a crucial role in promoting children’s health, they ought to embrace EBP so as to provide more effective services. School organization should play a supportive role with regard to EBP implementation.

## 1. Introduction

Evidence-based practice (EBP) has become the standard for provision of the best patient care in the clinical care system [1]. EBP as a foundation can help nurses clarify clinical problems, search for current research, and carry out evidence-based decision-making to solve those problems. The ultimate goal of adopting EBP is to improve the quality and effectiveness of care [2]. To increase EBP engagement among nurses in other setting, intense support, educational training and leadership is needed [3]. School nurses are expected to incorporate EBP into their daily routines and into their decision-making process. Available evidence shows that school nurses work across education and health with the task of meeting the increasingly complex health needs of children and their families [4,5,6]. To meet these increasingly diverse and complex needs, school nurses must reexamine their practice and demonstrate nursing practice that is based on the best evidence, to ensure efficiency in care and best outcomes for children [7]. 

In Taiwan, employment of EBP is a core competency for staff nurses. Therefore, many hospitals emphasize acquisition of the knowledge and skills needed for EBP through nursing educational programs [8,9,10,11,12]. However, little attention has been given to the use of EBP in the school nurse population [4,5,13]. More specifically, no research aiming to promote EBP among school nurses has been conducted in Taiwan [13]. Approximately 2644 school nurses care for more than one million school children in primary schools [14]. School nurses play a pivotal in the provision of routine day-to-day healthcare to all children, and also manage the complex health needs of many of these children [15,16]. The role of school nurses has expanded and become increasingly diverse over the past few decades. Taiwanese children are experiencing severe conditions such as obesity (29.7%) and myopia (61.8%), with these making up the highest rates in Asia [17,18,19]. Meanwhile, the Ministry of Education in Taiwan [20] has developed a school-based health promotion program and functions across different settings; for a given community, the school is an important setting within the broader system of the local environment. The school nurse is a key person in the programs to work with students, school staff, and parents to address health-related concerns and/or problems [21]. School nurses work towards creating a supportive, healthy school environment and connecting school community members with support services inside and outside the school [6,22]. School nurses are expected to be knowledgeable in managing children’s diverse and complex needs in the provision of health education and prevention programs in schools [16]. Taiwanese studies posit that EBP implementation in school settings can greatly improve the health outcomes of children, adolescents, and their families, and ultimately improve the quality of school nursing practice [23,24]. However, school nursing has been slow to implement EBP in Taiwan.

Several studies investigating nurses’ perceptions show that nurses hold positive views and attitudes toward EBP and consider it essential in the provision of quality care [25,26,27,28]. However, many barriers to the adoption of EBP in nursing have been identified [2,25,28,29,30]. Those consistently reported are inadequate research resources and lack of time, authority to change, skills to find and critically evaluate empirical research, and administrative support [2,10,30,31,32]. A significant barrier often cited for poor participation in EBP is limited EBP-related education and experience, particularly the incorporation of evidence into practice, which is the major reason for the small number of staff nurses who implement EBP [12,26,30,33,34]. Adib-Hajbaghery [25] investigated Iranian nurses’ perceptions and they perceived EBP as essential professional knowledge. However, Iranian nurses lacked the self-confidence to apply EBP strategies in clinical practice. Studies have identified associations between nurses’ self-confidence and EBP knowledge, skills, and administrative support [25,29,34].

Another significant barrier to EBP implementation in Taiwan is the limited ability to read research reports in English [34]. The best research evidence is published in English, which poses a big challenge for nurses in Taiwan [8,9,35,36]. When promoting EBP in school nursing practice, school nurses’ engagement in EBP must consider not only the above-mentioned barriers but also individual factors such as nurses’ age, duration of nursing practice, specialty, and experience of participating in research and EBP training [2,37]. Understanding the perceptions and factors relating to school nurses’ engagement in EBP can help strengthen EBP in the field of school nursing. This study aims to examine primary school nurses’ perceptions regarding EBP and to explore the factors that influence EBP in Taiwan.

## 2. Materials and Methods 

### 2.1. Study Design

A descriptive, cross-sectional design was used and a questionnaire survey was conducted in Taiwan between January and May 2014. This study was approved by The Ethical Review Committee of Chang Gung Hospital, Linkou, Taiwan (approval number: 103-3678C). All participants provided written informed consent prior to participation in the study. 

### 2.2. Participants

A total of 2679 elementary school nurses in 2013 in Taiwan at the time of the study [38] that met the criteria for inclusion were invited to participate in this study. School nurses who did not work in elementary schools were excluded. 

### 2.3. Strategies for Entry into Research Site

Approval for the study was given by the Ministry of Education in Taiwan and the Association of Chinese School Health Nursing. A list of school nurse in each county and city was supplied by the Association of Chinese School Health Nursing. The letter seeking permission and the consent form were sent and subsequently signed by the Ministry of Education and the Association of Chinese School Health Nursing. A formal government document was sent by the Ministry of Education to inform every elementary school nurse that the study had official support. Multiple data collection strategies were adopted to help achieve a better response, combining email, postal and web-based survey. A web link to the questionnaire was posted on the homepage by the Association of Chinese School Health Nursing. Afterwards, the questionnaire was email to all elementary school nurses with an information letter describing the purpose of study. Approximately two weeks, nurses received another email encouraging those who had not responded to do so as soon as possible. A copy of the questionnaire and a stamped, pre-addressed return envelope was sent in a third invitation.

### 2.4. Measure

The school nurses’ knowledge, attitudes, skills, self-efficacy, and influencing factors relating to EBP were measured with the School Nurse Evidence-Based Practice Questionnaire. The questionnaire was developed in Mandarin based on a comprehensive systematic review, following interviews with 75 elementary school nurses who had been randomly selected and a panel of 12 experts (8 academics in school health and 4 senior school nurses with training in EBP). The questionnaire was used back-translation strategy for checking translation accuracy for English reader in this paper. 

The questionnaire comprises six sections on demographic data, knowledge, attitudes, skills, self-efficacy, and influencing factors relating to EBP. The demographic data included age, education level, duration of employment as a school nurse, participation in research programs and EBP training courses, frequency of reading journal articles, and school size. According to the Ministry of Education in Taiwan [11], there are 3 types of schools, small, medium and large. A small school has 12 classes or less; a medium school has 13 to 48 classes; and a large school had more than 48 classes. Primary schools in Taiwan typically comprise classes of approximately 20–25 learners.

The knowledge section comprises 5 true/false/“I do not know” items. An incorrect or “I do not know” answer for an item is assigned a score of 0 and a correct answer, 1. Higher scores indicate higher levels of EBP knowledge. The attitudes, self-efficacy, and influencing factors sections each comprise 12 items, and the skills section, 10 items. All items are responded to on a 5-point Likert-scale ranging from 1 (completely disagree/no confidence) to 5 (completely agree/complete confidence). Higher scores indicate better attitudes, skills, self-efficacy, and perceptions relating to EBP. All negatively worded items in the questionnaire were reverse-coded.

### 2.5. Validity and Reliability

The validity and reliability of the questionnaire were assessed. The content validity and face validity of the EBP questionnaire were verified by computing the Content Validity Index (CVI), using ratings of item relevance by a panel of 12 experts. No item was eliminated in the CVI assessment, and all items had a score above 0.78. Only six items were revised to be more appropriate. The preliminary questionnaire was piloted with 150 elementary school nurses. Cronbach’s alpha coefficient was used to evaluate the stability and internal consistency of the instrument. Cronbach’s alpha’s coefficients were reported to be more than 0.72, indicating a high level of internal consistency in each section of this questionnaire (knowledge: α = 0.72; attitudes: α = 0.78; skills: α = 0.89, self-efficacy: α = 0.94; and influencing factors: α = 0.89).

Factor analysis was used to examine construct validity. The attitudes, skills, self-efficacy, and influencing EBP factors scales were subjected to factor analysis. Oblique rotation was used to approach the results. The 52 items in the first draft of questionnaire were entered into four factors to analysis. Examination of the scree plot indicated the presence of one factor explaining 50.77% of the variance. Factor loadings for items were greater than 0.3 and loaded in the same component suggesting that items made an important contribution in the subscale. In the final version of questionnaire, 46 items were tested.

### 2.6. Statistical Analysis

Statistical analysis was performed using IBM SPSS Statistics for Windows, Version 23 (SPSS Inc., Chicago, IL, USA). Descriptive statistics (percentages of frequencies, means, and standard deviations) were calculated to describe the distribution of responses for each item. To examine the associations of various background variables with variables on subscales (knowledge, attitudes, skills, self-efficacy, and influencing EBP factors) we used Pearson’s correlation coefficient. Background variables selected for the analyses included age, education level, continuing education, duration of employment as a school nurse, frequency of reading journal articles, participation in research programs and EBP training courses, and school size, respectively. *P* values less than 0.05 were considered statistically significant for all tests.

## 3. Results

### 3.1. Participant Characteristics

A total of 2679 elementary school nurses in Taiwan were invited to participate in this study; 1200 completed questionnaires were returned, resulting in a response rate of 45%. All participants were female. Participants’ mean age was 37.5 years (range: 31–62) years. The mean duration of employment as a school nurse was 8.4 (range: 1–20) years. Table 1 shows participants’ demographic data. 

Table 2 presents participants’ attendance of an in-service training course within the previous year. The top three training courses in the previous year were a health promotion program, infectious disease prevention and management, and emergency care.

### 3.2. School Nurses’ Knowledge, Attitudes, Skills, Self-Efficacy, and Influencing Factors of Implementing EBP 

The mean score in the knowledge section was 2.88 (SD = 1.08), indicating that school nurses had insufficient EBP knowledge. The item with the highest score (94.7%) was “The concept of EBP means helping nursing staff identify problems and develop problem-solving abilities,” followed by “Five steps of EBP” (41.3%). The item with the lowest score (10.7%) was “When conducting meta-analysis, one of the goals is to draw conclusions from relevant studies,” followed by “Level of evidence” (17.3%) (Table 3).

The mean score in the attitude section was 3.60 (SD = 0.43), indicating that school nurses had a positive attitude towards EBP. However, the item with the lowest score was “Lack of time to implement EBP” (mean = 2.31, SD = 1.06), showing that school nurses believed that EBP would increase service duration (Table 4).

The mean score in the skill section was 2.80 (SD = 0.54), indicating that school nurses perceived themselves as having insufficient skills in EBP. Only items 9 (“I will think about common student health issues in school”) and 10 (“I will search for relevant literature on the Internet to solve student health issues”) yielded average scores greater than 3.5. The scores for other items were all lower than 3 (mean: 2.40–2.73), indicating that school nurses had insufficient skills in EBP (Table 5).

The mean score in the self-efficacy section was 3.20 (SD = 0.74), showing that school nurses had moderate confidence in implementing EBP. The item associated with the lowest level of confidence was the ability to read English research articles (mean = 2.76, SD = 1.01). It is worth noting that the average scores on items 7, 9, 10, and 12 were all lower than 3, and these items were related to application of EBP in the workplace (Table 6).

The mean score in the influencing factors section was 2.77 (SD = 0.44). This shows that school nurses believed that there were many factors affecting the implementation of EBP in schools. School nurses indicated that the lowest factor to influence EBP in schools were “most research articles are published in English and are difficult to understand” (mean = 2.31, SD = 0.99). Other factors such as the lack of relevant skills, support systems, and relevant resources are also important to consider when implementing EBP in schools (Table 7).

### 3.3. The Correlations between Demographic Data, Knowledge, Attitudes, Skills, Self-Efficacy, and Influencing Factors for Implementing EBP

Table 8 shows the bivariate Person’s correlations between independent demographic data and knowledge, attitudes, skills, self-efficacy and influencing factors for implementing EBP in the School Nurse Evidence-Based Practice Questionnaire. School nurses’ knowledge, skills, and self-efficacy positively and significantly correlated with education level, continuing education, frequency of reading journal articles, participation in research programs and EBP training courses (*r* = 0.07~0.33, *p* < 0.05). The influencing factors positively and significantly correlated with age, education level, continuing education, frequency of reading journal articles and EBP training courses (*r* = 0.09~0.20, *p* < 0.05).

## 4. Discussion

This study investigated primary school nurses’ perceptions of and to explore the factors that influence EBP in Taiwan. Our results showed that school nurses have insufficient EBP knowledge. Most answered the first question correctly (94.7%), which is related to the concept of EBP, but less than 50% answered questions correctly on aspects such as EBP steps and evidence level which is EBP basic knowledge. Further, our study found that school nurses have insufficient EBP skills, such as utilizing the PICO framework, accessing EBP databases, and critical appraisal of articles. Critical appraisal and evaluation of research evidence in articles are difficult steps in EBP; this is also the greatest obstacle to implementation of EBP by clinical staff [12,26,30,33,34]. Although EBP is one of the core competences in clinical practice, there remains a need to strengthen relevant EBP knowledge and skills [10,25]. Many studies have indicated that education and training can improve nursing staff’s EBP knowledge and skills, and aid in the implementation of EBP [9,10,12,39,40,41]. Conversely, this study found that only 5.3% of school nurses had attended EBP training in the previous year. Therefore, it is necessary and urgent to design a tailored EBP training course for school nurses.

In our study, school nurses had positive attitudes towards EBP and perceived it to be important and beneficial in schools, with the potential to improve the quality of healthcare for students. However, school nurses believed that EBP implementation would increase their workload. This result is similar to those of other studies [26,30,42]. Other research has demonstrated that, not only can educational interventions affect nurses’ knowledge and skills, but they can also enhance their attitudes toward EBP and result in greater engagement in it [39]. Our study found that school nurses have moderate self-efficacy in implementing EBP, but lower self-efficacy in integrating evidence, and reading English articles. Research articles are published mainly in English. The stress and frustration stemming from reading the literature may be one of the reasons for the low frequency of journal-reading by school nurses [35]. Lee [43] examined 1042 nurses’ database usage and found that they used English databases less frequently. This may also be due to their limited ability to read English articles that limited their use of databases. Therefore, understanding school nurses’ self-efficacy can not only help in clarifying their level of confidence regarding implementation of each EBP step but also be used to evaluate the effects of evidence-based healthcare services.

In this study, the factors influencing EBP implementation include knowledge, skills, time constraints, inability to understand English articles, lack of support, and lack of relevant resources. These are consistent with those in a number of studies [2,25,28,33,34,44]; in future, these factors should be considered collectively when implementing EBP in schools. Our results regarding EBP knowledge, as well as skills and self-efficacy in EBP implementation showed significant positive correlations with education level, continuing education, the frequency of reading journals, and participation in research programs and in EBP training courses among school nurses. This suggests that school organizations must establish a series of structured educational interventions to enable the application of EBP.

## 5. Conclusions

This study investigated school nurses’ knowledge, attitudes, skills, self-efficacy, and influencing factors relating to EBP. Although school nurses had a positive attitude and moderate self-efficacy, their EBP knowledge and skills were insufficient. Certain factors influencing EBP implementation include knowledge, skills, time constraints, inability to understand English articles, lack of support, and lack of relevant resources. As school nurses play a crucial role in promoting children’s health, they ought to embrace EBP so as to provide more effective services. Moreover, school organizations should play a supporting role in relation to EBP implementation. The results of this study can be used as a basis for the development of a comprehensive strategy for building EBP competencies through proper training.

## Figures and Tables

**Table 1:** Participants’ demographic data (*N* = 1200).

Variables	*N*	%
Highest degree		
Master’s degree	208	17.3
Bachelor’s degree	672	56.0
Diploma	320	26.7
Size of school		
Large (more than 48 classes)	304	25.3
Medium (13–48 classes)	576	48.0
Small (12 classes or less)	320	26.7
Continuing education		
No	976	81.3
Yes	224	18.7
Participation in research programs		
No	816	68.0
Yes	384	32.0
Participation in EBP training courses		
No	970	81.0
Yes	230	19.0
Frequency of reading journal articles in the previous year		
Never	192	16.0
Several months	544	45.3
At least every month	288	24.0
Every two weeks	80	6.7
At least every week	96	8.0
Motivation for attending EBP training (Multiple choice)		
Job requirements	1008	84.0
Personal interests	528	44.0
Nursing continuing education requirements	288	24.0
Organizational expectations	240	20.0

**Table 2:** Attending an in-service training course in the previous year (multiple choice) (*N* = 1200).

Courses	*N*	%	Ranking
Health promotion program	992	82.7	1
Infectious disease prevention and management	976	81.3	2
Emergency care	944	78.7	3
Health information management	608	50.7	
Prevention and resolution legal disputes	544	45.3	
Crisis management	464	38.7	
Health center management	352	29.3	
Health assessment	288	24.0	
Education activities design and planning	128	10.7	
School−community collaboration	64	5.3	
Evidence-based practice	64	5.3	
Individual health counseling	48	4.0	
Nursing research	16	1.3	
Statistics	16	1.3	

**Table 3:** Knowledge of EBP (*N* = 1200).

Item Content	*N*	(%)
1. The concept of EBP means helping nursing staff identify problems and develop problem-solving abilities.		
No points awarded	64	5.3
Points awarded	1136	94.7
2. Five steps of EBP are as follows: (1) Ask a question, (2) Find information/evidence to answer question, (3) Integrate appraised evidence with own clinical expertise and patient’s preferences, (4) Critically appraise the information/evidence, and (5) Evaluate.		
No points awarded	704	58.7
Points awarded	496	41.3
3. When conducting meta-analysis, one of the goals is to draw conclusions from relevant studies.		
No points awarded	1072	89.3
Points awarded	128	10.7
4. In the PICO framework, the “C” represents “chief complaint.”		
No points awarded	800	66.7
Points awarded	400	33.3
5. In the 5 levels of evidence, the lower the score, the higher the level of evidence.
No points awarded	992	82.7
Points awarded	208	17.3
The Knowledge Section	Mean	SD
	2.88	1.08

**Table 4:** Attitudes regarding EBP (*N* = 1200).

Item Content	Mean	SD
The Attitude Section	3.60	0.43
1. When I identify problems in school, I am willing to adopt an EBP process to solve them.	3.74	1.15
2. The application of EBP in my work can improve the quality care of students.	4.12	0.67
3. EBP can be used to solve school problems.	4.04	0.68
4. The steps in EBP can assist my professional growth.	4.13	0.66
5. EBP can be used as practical guidelines for school nursing.	4.01	0.78
6. Implementing EBP will increase my workload.	3.34	1.17
7. There is a lack of time to implement EBP.	2.31	1.06
8. Implementing EBP will make student health services more complicated.	3.28	1.10
9. EBP is only theory and cannot be applied in nursing care in schools.	3.47	0.96
10. Medical professionals should be equipped with EBP knowledge and skills.	3.60	1.31
11. Engaging in EBP enabled me to perceive the value of and feel confident in school nursing work.	3.87	0.79
12. I will use evidence-based information to discuss student health issues with colleagues/students/parents.	3.28	1.33

**Table 5:** Skills in EBP (*N* = 1200).

Item Content	Mean	SD
The Skill Section	2.80	0.54
1. I can utilize the PICO framework to create a researchable question related to school of nursing.	2.56	1.23
2. I know how to access EBP databases.	2.65	1.07
3. I can critically appraise articles.	2.56	1.03
4. I can evaluate the level of evidence in an article.	2.40	1.01
5. I can select articles with the highest level of evidence from relevant studies.	2.51	1.08
6. I can carry out a literature search and obtain data within 30 minutes.	2.73	0.90
7. I can summarize the results of multiple articles to guide healthcare decisions that are most beneficial to student.	2.52	1.43
8. I will apply EBP in school.	2.63	1.28
9. I will think about common student health issues in school.	3.75	0.75
10. I will search for relevant literature on the Internet to solve student health issues.	3.67	0.84

**Table 6:** Self-efficacy in implementing EBP (*N* = 1200).

Item Content	Mean	SD
The Self-efficacy Section	3.20	0.74
1. I can routinely ask questions at work.	3.77	0.65
2. My workplace can assist me in understanding research articles related to my work.	3.22	0.94
3. I can promote EBP in my workplace.	3.24	0.85
4. I can critically appraise articles based on school expertise and clients’ values to guide healthcare decisions.	3.14	1.22
5. I can obtain support from my workplace/organization to implement EBP.	3.23	1.00
6. I can routinely inspect the health outcomes of students.	3.65	0.92
7. I can integrate evidence and apply it in school nursing.	2.86	1.25
8. I can apply EBP in my workplace.	3.25	1.04
9. I can routinely evaluate literature in my workplace.	2.92	1.17
10. I can routinely implement the EBP process in my workplace.	2.91	1.34
11. I can participate in EBP training courses.	3.61	1.04
12. I can read English research articles.	2.76	1.01

**Table 7:** Influencing factors in implementing EBP (*N* = 1200).

Item Content	Mean	SD
The Influencing Factors Section	2.77	0.44
1. School nurses lack EBP knowledge.	2.32	0.84
2. School nurses lack EBP skills.	2.47	0.91
3. School nurses lack appropriate peer influence or role models.	2.39	0.91
4. There is insufficient time of implementing EBP at work.	2.33	0.99
5. Most research articles are published in English and are difficult to understand.	2.31	0.99
6. Only school nurses with a university-level education and higher have the ability to implement EBP.	4.14	0.76
7. It is difficult to apply research evidence in school nursing.	2.57	1.04
8. I do not have any right to change the current school system.	3.02	1.34
9. I lack support from school administrators.	2.50	0.98
10. I lack Internet resources such as medical and nursing databases.	2.94	1.34
11. I lack computers.	4.09	0.72
12. I am unable to obtain expert guidance and aid when implementing EBP.	2.40	0.96

**Table 8:** The correlations between demographic data, knowledge, attitudes, skills, self-efficacy, and influencing factors *(N* = 1200).

Item	Knowledge	Attitudes	Skills	Self-Efficacy	Influencing Factors
Age	−0.03	0.10 **	0.02	0.05	0.13 **
Education level	0.20 **	0.11	0.30 **	0.18 **	0.20 **
Continuing education	0.18 **	0.09 *	0.17 *	0.07 *	0.15 **
Duration of employment as a school nurse	−0.01	0.14 *	0.06 *	0.06 *	0.02
Frequency of reading journal articles	0.19 **	0.21	0.24 **	0.16 **	0.16 **
Participation in research programs	0.28 **	0.07 **	0.18 **	0.16 **	−0.00
Participation in EBP training courses	0.33 **	0.01	0.31 **	0.13 **	0.09 **
School size	0.22 **	−0.20 **	0.10 **	−0.04	−0.18 *

* *p* < 0.05, ** *p* < 0.01
